# TDP‐43 Phosphorylation: Pathological Modification or Protective Factor Antagonizing TDP‐43 Aggregation in Neurodegenerative Diseases?

**DOI:** 10.1002/bies.70084

**Published:** 2025-11-02

**Authors:** Simone Mosna, Dorothee Dormann

**Affiliations:** ^1^ Institute of Molecular Physiology Faculty of Biology Johannes Gutenberg University Mainz Germany; ^2^ International PhD Programme (IPP) of IMB Mainz Mainz Germany; ^3^ Institute of Molecular Biology (IMB) Mainz Germany

**Keywords:** amyotrophic lateral sclerosis (ALS), condensates, frontotemporal dementia (FTD), kinases, neurodegeneration, phase separation, phosphorylation, protein aggregation, TAR DNA binding protein of 43 kDa (TDP‐43)

## Abstract

TDP‐43 is a ubiquitously expressed RNA‐binding protein that aggregates in the brains of patients suffering from neurodegenerative diseases, such as amyotrophic lateral sclerosis (ALS), frontotemporal dementia (FTD) and Alzheimer's disease. Aggregated TDP‐43 in these diseases is hyperphosphorylated in its C‐terminal intrinsically disordered region, while physiological TDP‐43 is normally unphosphorylated. Whether TDP‐43 phosphorylation is a pathological driver, or rather a protective antagonist of TDP‐43 aggregation and consequently neurodegeneration, is still debated and a matter of ongoing research. Here, we review current knowledge about TDP‐43 phosphorylation in disease and the kinases and phosphatases that regulate this post‐translational modification. We discuss how TDP‐43 phosphorylation is thought to shape TDP‐43's phase separation, aggregation and toxicity in neurodegenerative diseases. We highlight recent research that provides evidence that hyperphosphorylation antagonizes TDP‐43 phase separation and aggregation, and speculate about a potential role of condensates in TDP‐43 phosphorylation.

## Graphical abstract

TDP‐43 hyperphosphorylation occurs in neurodegenerative diseases, however, it is not a driver of TDP‐43 aggregation, but enhances TDP‐43 solubility, suggesting that TDP‐43 hyperphosphorylation is protective. Toxicity may arise from a broader phosphorylation‐driven cellular pathway disruption. Inhibiting kinases may thus restore neuronal health indirectly, rather than by modulating TDP‐43 aggregation. Created in BioRender. Dormann, D. (2025) https://BioRender.com/qp169wg

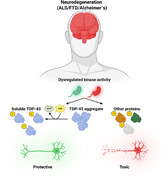

## 1 Introduction

Neurodegenerative Diseases, such as Alzheimer´s disease (AD), Parkinson disease (PD), amyotrophic lateral sclerosis (ALS) and various forms of dementia, lead to a range of cognitive and physical impairments and pose significant challenges for the elderly. These conditions often feature inclusion bodies made of aggregated proteins in neuronal cells, which can spread through prion‐like mechanisms across the nervous system [1].

A key protein that gets deposited in inclusions in some of these diseases is TDP‐43 (TAR DNA binding protein of 43 kDa). TDP‐43 aggregates are prevalent in more than 90% of ALS patients, ∼50% of frontotemporal dementia (FTD) patients, up to 60% of AD cases, as well as a more recently recognized dementia called limbic‐predominance age‐related TDP‐43 encephalopathy (LATE) [2, 3, 4]. TDP‐43 is a DNA/RNA‐binding protein normally found in the nucleus of all cells. It plays important roles in DNA/RNA processing, such as the regulation of transcription, alternative splicing, microRNA biogenesis, translation, mRNA transport and DNA damage repair [5, 6, 7]. In the above mentioned diseases, however, TDP‐43 redistributes from the nucleus to the cytoplasm and forms cytoplasmic aggregates in neurons and glial cells [4, 8]. These aggregates, composed of full‐length TDP‐43 and truncated C‐terminal fragments, are harmful as they trap TDP‐43 away from the nucleus, impairing its vital roles in RNA processing and regulation [5, 6, 7]. This nuclear loss‐of‐function, possibly combined with direct toxic gain‐of‐functions of the cytoplasmic aggregates, impairs neuronal function and eventually leads to neurodegeneration.

How TDP‐43 forms pathological aggregates is still a very current research topic. ALS and FTD patients feature mixtures of amorphous, non‐amyloid TDP‐43 aggregates as well as amyloid‐like filaments whose ordered core structures have recently been resolved by cryo‐electron microscopy (cryo‐EM) [9, 10, 11, 12, 13]. Through which pathway(s) these amorphous and amyloid‐like filaments form is still unclear. TDP‐43 has extended intrinsically disordered regions (IDRs), which make the protein inherently prone to self‐assembling into oligomers or larger protein droplets through the process of phase separation [14, 15, 16]. These self‐assembly pathways may play important roles in the formation of both the amorphous TDP‐43 aggregates and the amyloid‐like TDP‐43 fibers [17, 18]. Phase separation refers to a condensation process where a homogenous solution of molecules separates into two distinct phases—a dense phase and a dilute phase, reminiscent of oil droplets forming in water [19]. Proteins with IDRs of low sequence complexity have a high tendency to undergo phase separation, as these regions mediate multivalent interactions, which are critical for phase separation. TDP‐43 contains a long C‐terminal low complexity region (LCR), which is largely disordered but harbors a conserved α‐helical element [15, 16]. In particular, aromatic and aliphatic residues in the LCR, as well as the α‐helix, contribute to TDP‐43's phase separation into droplet‐like structures [15, 16, 20]. While phase separation is a regulated and reversible process under normal conditions, various factors, such as increased protein concentration or disease‐linked mutations, can enhance the propensity of proteins within condensates to misfold and give rise to solid, aggregated or amyloid‐like states [14, 21, 22]. Moreover, the condensate surface can promote transitions to misfolded cross‐beta structures and nucleate amyloid fibers of various disease‐linked proteins [23, 24, 25, 26]. Understanding how cells regulate such aberrant phase transitions from a dynamic, liquid‐like condensed state to a solid, aggregated state is crucial for developing therapeutic strategies to prevent or reverse pathological protein aggregates.

Post‐translational modifications (PTMs), such as phosphorylation, acetylation, SUMOylation and ubiquitination, often regulate protein‐protein interactions, and hence they can influence the propensity of proteins to undergo phase separation and transition to aggregates [27, 28, 29]. TDP‐43 aggregates in ALS and FTD patients show significant phosphorylation and poly‐ubiquitination [4], unlike TDP‐43 in healthy individuals. Therefore, these PTMs are heavily researched for their role in disease.

This essay will discuss TDP‐43 phosphorylation, examining various hypotheses about its occurrence in disease, by which kinases it is introduced, and, in particular, what consequences it has on TDP‐43 aggregation. By this, we pick up some of the ongoing debates in the scientific community about the relevance of TDP‐43 phosphorylation and TDP‐43 kinases for pathology and potential treatment of neurodegenerative disorders.

## 2 TDP‐43 Hyperphosphorylation in Neurodegenerative Diseases

**FIGURE 1:**
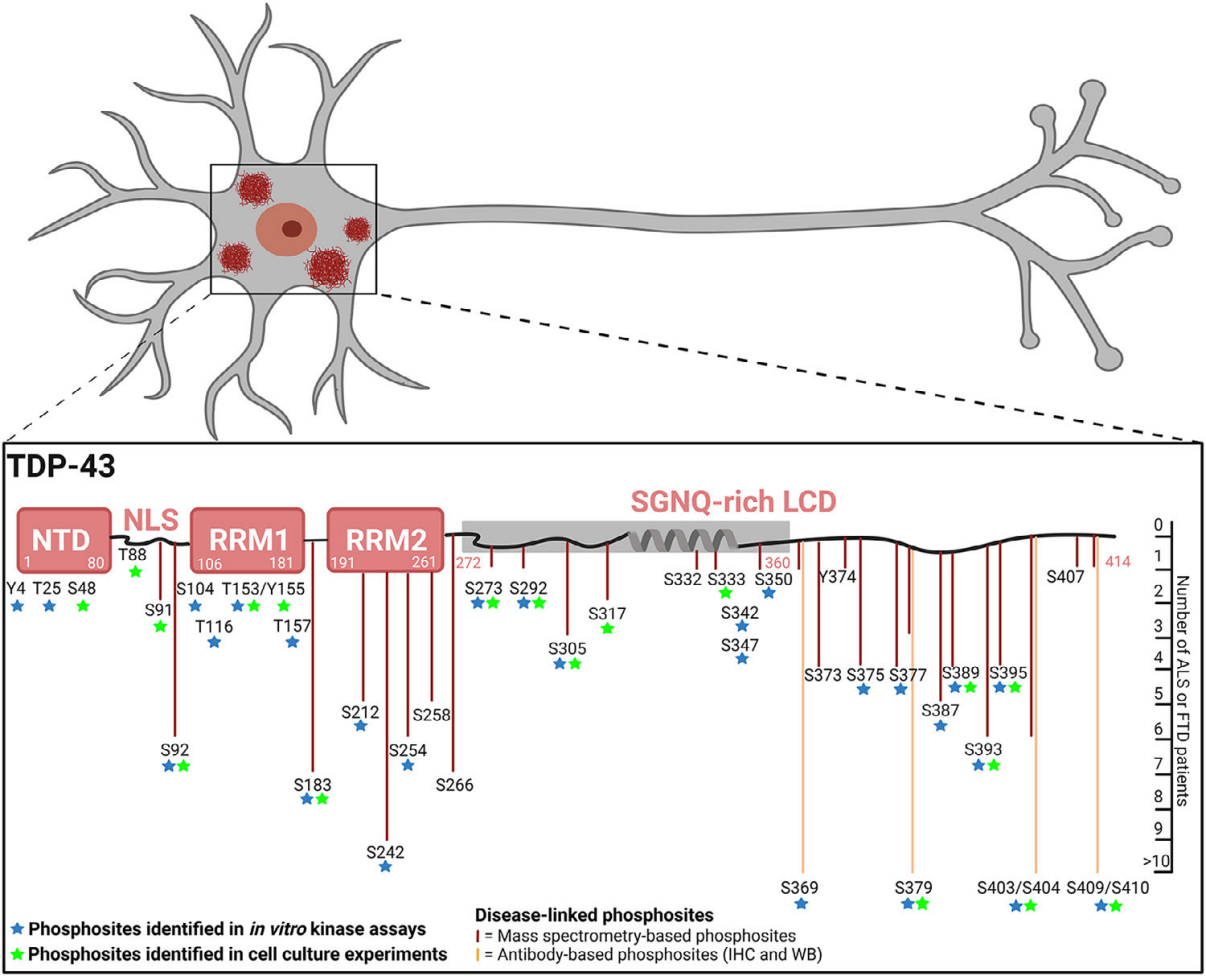
TDP‐43 phosphorylation sites identified in post‐mortem tissue of patients or cell culture studies. TDP‐43 contains a folded N‐terminal domain (NTD), a short disordered region harboring a nuclear localization signal (NLS), two tandem RNA recognition motif (RRM) domains and an LCD enriched in serine, glycine, asparagine, and glutamine (SGNQ) residues. The shaded region indicates the protease‐resistant amyloid‐like core of TDP‐43 aggregates identified by recent cryo‐EM structures [11, 12, 13]. Phospho‐sites identified in patients are shown below the scheme, the length of the lines indicates in how many patients they were identified. Dark red lines represent phospho‐sites identified from unbiased MS [39, 40], whereas orange lines represent phospho‐sites identified through antibody‐based immunohistochemistry (IHC) or Western blot (WB) [4, 30, 31, 32]. Phospho‐sites identified in various cell culture studies are marked with a green asterisk [41, 42, 43, 44, 45, 46], and the ones identified in in vitro kinase experiments are marked with blue asterisks [30, 47, 48] (from PhosphoSitePlus [49]). Created in BioRender. Dormann, D. (2025) https://BioRender.com/pyomoev.

TDP‐43 contains numerous potential phosphorylation sites (41 serines, 15 threonines, and 8 tyrosines), among them many serines in the LCR. TDP‐43 Western blots on insoluble brain material from ALS or FTD patients show, in addition to a 43 kDa band, a 45 kDa band, which by dephosphorylation collapses into the 43 kDa band [4]. This migration behavior indicates the presence of multiple phospho‐groups on at least a portion of the TDP‐43 molecules in the disease state. Hence, TDP‐43 was described early on as being “hyperphosphorylated” in disease conditions. Phospho‐specific antibodies have identified a few key sites that are typically phosphorylated in TDP‐43 aggregates in neurodegenerative conditions, these include C‐terminal serines S369, S379, S403/S404, and S409/S410 [30, 31, 32] (Figure 1, orange lines). Such phospho‐specific antibodies, in particular those specific to phospho‐S409/S410, are routinely used to detect TDP‐43 pathology in post‐mortem tissue of patients [33]. Most studies of patient tissue show phosphorylated TDP‐43 at S403/S404 and S409/S410 in neuronal and glial cytoplasmic inclusions in FTD and AD patients [31, 34], as well as in motor neurons in ALS patients [31, 35]. Furthermore, phosphorylated TDP‐43 has also been detected in non‐neuronal cells, such as astrocytes, in FTD, AD and LATE patients [36, 37, 38]. In addition, unbiased mass spectrometry (MS)‐based phospho‐proteomics has revealed multiple phosphorylation sites in the C‐terminal LCR as well as more N‐terminal regions in a small cohort of ALS and FTD patients [39, 40] (Figure 1, dark red lines), expanding the view of TDP‐43's phosphorylation landscape. However, only small patient numbers (two ALS [40], five FTD‐TDP type A and two FTD‐TDP type B cases [39]) have been examined so far, and these studies did not reveal the total phosphorylation level across TDP‐43 molecules, hence it is still unclear which fraction of TDP‐43 molecules is truly “hyperphosphorylated” in disease, since phosphosite occupancy can only be quantitatively determined through targeted proteomics. Moreover, there is very limited information on the phosphorylation status of TDP‐43 in cellular or animal models, such as iPSC‐derived neurons or mouse models of TDP‐43 proteinopathy—typically only the presence of S409/S410 is probed, while comprehensive information on TDP‐43 phospho‐sites (number, location, stoichiometry) is largely missing. Non‐demented control brains (without TDP‐43 pathology) usually show no signal with any of the phospho‐specific antibodies [30, 31, 32], and in three examined control cases, no phospho‐peptides were identified by MS [39].

Nevertheless, various studies have identified phosphorylation sites in TDP‐43 under different experimental conditions, as evident from public databases, such as PhosphoSitePlus or dbPTM, which list additional phosphorylation sites so far not linked to disease but detected by MS‐based phospho‐proteomics or other methods in human cancer cell lines (Figure 1, green stars). For example, T153/Y155 phosphorylation was found upon heat shock in HEK293T and SH‐SY5Y cells [41], and T88, S91/S92 phosphorylation in TDP‐43's nuclear localization signal (NLS) in HeLa cells upon EGF stimulation, in breast cancer cell lines as well as melanoma cells upon MKK1/2 inhibitor treatment, possibly to regulate TDP‐43's nuclear import [31, 42, 43, 44, 45]. Moreover, additional phospho‐sites were found by MS on recombinant TDP‐43 phosphorylated in vitro by purified Casein kinase 1 (CK1) [47, 48] (Figure 1, blue stars), however, it is unclear to what extent such in vitro kinase reactions reflect the physiological or pathological situation. The fact that several phosphorylation sites were reported in different human cell lines under normal conditions suggests that phosphorylation may be a dynamic signal to regulate TDP‐43's function. Hence, it is possible that disease‐specific phospho‐sites may arise from aberrant cellular processes that involve TDP‐43 phosphorylation as part of physiological regulatory mechanisms.

Interestingly, different disease subtypes show differential recognition by a pS369‐specific antibody in immuno‐histochemistry (ALS‐TDP, FTD‐TDP type B and C cases showed strong immunoreactivity, while FTD‐TDP type A cases were negative) [32]. In contrast, under denaturing conditions, all subtypes were recognized by the same antibody, suggesting that TDP‐43 inclusions in different subtypes of TDP‐43 proteinopathies adopt different structural conformations [32]. This is in line with the recent cryo‐EM structures of brain‐derived TDP‐43 aggregates, which revealed that the protease‐resistant core of the extracted TDP‐43 aggregates has a different amyloid‐like fold in ALS and FTD‐TDP type B cases than in FTD‐TDP type A cases [12, 13]. Interestingly, most serine residues in the amyloid‐like core of TDP‐43 are buried; hence, it remains to be determined whether disease‐associated phosphorylation of these sites would even be compatible with the formed structures. Notably, most phospho‐sites detected in ALS and FTD patients reside outside the amyloid‐like core region (in the NLS‐RRM1‐RRM2 regions or very C‐terminal region (aa. 361‐414) (Figure 1). It would be highly informative to extend studies of TDP‐43 phosphorylation to larger patient cohorts, as well as all disease subtypes (ALS, FTD‐TDP‐43 type A, B, C and D, AD with TDP‐43 pathology and LATE), and to correlate them with structural approaches, such as cryo‐EM or structural proteomics approaches. In a related class of neurodegenerative disorders caused by the deposition of the Tau protein (AD and corticobasal degeneration, CBD), different Tau structures have indeed been correlated with different PTM patterns, suggesting that PTMs influence the Tau filament structure [50]. Similarly, it can be speculated that the TDP‐43 phosphorylation pattern may influence the TDP‐43 amyloid‐like fold. Moreover, it remains to be investigated whether TDP‐43 phosphorylation influences the formation of amorphous, non‐amyloid TDP‐43 aggregates that have also been observed in ALS and FTD patient brains [9, 10].

How could different TDP‐43 phosphorylation patterns arise? It seems possible that different kinases/phosphatases and signaling pathways become activated under different conditions or in different cell types. This raises the question of which kinases and phosphatases act on TDP‐43 and under which conditions they start phosphorylating the protein, in particular in disease‐relevant cell types.

## 3 Kinases and Phosphatases Linked to TDP‐43 Phosphorylation and Their Potential Misregulation in Disease

In the last 15 years, various kinases and phosphatases have been shown to act on TDP‐43 and regulate its phosphorylation status (Tables 1 and 2). They have been identified through various experimental approaches, most notable overexpression of candidate kinases or phosphatases and use of kinase/phosphatase inhibitors in cell or animal models, as well as in vitro phosphorylation/dephosphorylation assays with purified enzymes.

**TABLE 1:** Overview of proposed TDP‐43 kinases. Columns provide details on their links to TDP‐43 pathology, their subcellular localization and expression profile according to the Human Protein Atlas (www.proteinatlas.org) [70, 71], their protein abundance in parts per million (ppm) and how they rank against all the identified proteins according to the PaxDb [72, 73] protein abundance database integrated among multiple datasets in the brain [74, 75, 76, 77, 78], experimental evidence for their activity towards TDP‐43, and their links to phase separation and localization in membrane‐less organelles (MLOs) according to the RNA granule database [79].

TDP‐43 kinases	Links to TDP‐43 pathology in AD/ALS/FTD patients	Localization/expression [protein abundance in the brain in parts per million (ppm) and abundance rank position out of all the identified proteins]	Experimental evidence for activity towards TDP‐43	Links to phase separation and MLOs
CK1 kinases				
Casein kinase 1δ (CK1δ)	Overexpression at the protein level has been found in AD cases [63]	Nuclear and cytoplasmic localization, expression in all tissues with low tissue specificity [5.76 ppm, 4222. out of 13366]	Kinase assay with recombinant TDP‐43 (with S379, S403/S404 and S409/S410 phospho‐abs and with radio‐labeled ATP in WB and with MS) [31, 47, 48, 52, 59] In neuroblastoma SH‐SY5Y cells (with S379, S403/S404 and S409/S410 phospho‐abs in WB with S409/S410 phospho‐ab in IF and with MS) [45, 52, 59] and in mice (reduced S409/S410 signal after inhibition) [51] Directly interacts with TDP‐43 after IP (immunoprecipitation) from N2a cells [59]	Co‐localization with known markers of P bodies [80] Predicted to phase separate [79]
Casein kinase 1ε (CK1ε)	Overexpression at the RNA and protein level has been found in cases of sporadic ALS and AD [55, 60, 61]	Mainly cytoplasmic and nuclear localization, expression in several tissues with low tissue specificity [9.6 ppm, 3522. out of 13366]	Kinase assay with recombinant proteins (with radio‐labeled ATP) [52] In HEK‐293FT, U2OS and neuroblastoma SH‐SY5Y cells (with S379, S403/S404 and S409/S410 phospho‐abs in WB and with S409/S410 phospho‐ab in IF) [52, 55] and in mice (reduced S409/S410 signal after inhibition) [51] Directly interacts with TDP‐43 after IP from rat brain extract [55]	/
Tau‐tubulin kinase 1 (TTBK1)	Co‐localizes with phospho‐S409/S410 positive TDP‐43 aggregates in spinal cord neurons of ALS patients [57]	Cytoplasmic localization, expression in neuronal cells in the central nervous system (CNS) and in peripheral nerves [1.08 ppm, 6559. out of 13366]	Kinase assay with recombinant proteins (with S403/S404 and S409/S410 phospho‐abs in WB) [56, 57] In *C. elegans*, in NSC‐34 cells, Neuro2A cells, SH‐SY5Y cells and HEK293 cells (with S403/S404 and S409/S410 phospho‐ab in WB) [56, 57, 58]	/
Tau‐tubulin kinase 2 (TTBK2)	Co‐localizes with phospho‐S409/S410‐positive TDP‐43 aggregates in spinal cord neurons of ALS patients [57]	Cytoplasmic localization, expression in several tissues, mostly in cilia in retina, and in fallopian tube and sperm in testis [0.03 ppm, 11082. out of 13366, bottom 25%]	Kinase assay with recombinant proteins (with S403/S404 and S409/S410 phospho‐abs in WB) [56, 57] In *C. elegans*, in NSC‐34 cells, Neuro2A cells, SH‐SY5Y cells and HEK293 cells (with S403/S404 and S409/S410 phospho‐ab in WB) [56, 57, 58]	/
Other kinases				
Casein kinase 2 (CK2)	/	Nuclear and cytoplasmic localization, expression in most tissues with low tissue specificity [116 ppm, 1150. out of 13366, top 10%]	Kinase assay with recombinant proteins (with S379, S403/S404 and S409/S410 phospho‐abs in WB) [31, 81] In Neuro2A neuroblastoma cells (with S379, S403/S404 and S409/S410 phospho‐abs in WB) [82]	Found as interactor of C9orf72‐linked Dipeptide Repeat proteins in HEK293T cells by AP‐LC‐MS/MS [83]
Cell division cycle 7‐related protein kinase (Cdc7)	/	Nuclear localization, expression in most tissues, highly abundant in testis [NA]	Kinase assay with recombinant proteins (with S409/S410 phospho‐ab in WB) [64] In *C. elegans*, in human motor neuron enriched NSC‐34 cells and HEK293 cells (with S409/S410 phospho‐ab in WB) [64]	Found as modulator of MLO homeostasis based on an RNAi screen in HeLa cells [84] Predicted to phase separate [79]
Inhibitor of nuclear factor kappa‐B kinase subunit beta (IKβ)	/	Cytoplasmic localization, expression in most tissues with low tissue specificity [0.33 ppm, 8197. out of 13366]	Kinase assay with recombinant proteins (with S92 phospho‐ab in WB) [85] In HEK293T and in neuroblastoma Neuro2A cells (with S92 phospho ab in WB and in IF and with MS) [85]	/
CMGC kinases				
Mitogen‐activated protein kinase p38α (MAPK14, p38α)	/	Cytoplasmic and nuclear localization, expression with low tissue specificity [2.42 ppm, 5432. out of 13366]	Kinase assay with recombinant proteins (with S409/S410 phospho‐ab in WB) [86] In neuroblastoma SH‐SY5Y cells (with S409/S410 phospho‐ab in WB and in IF) [86] Directly interacts with TDP‐43 after IP from SH‐SY5Y cells [86]	/
STE kinases				
Dual specificity mitogen‐activated protein kinase kinase 1 (MAP2K1, MEK1)	/	Cytoplasmic localization, expression with low tissue specificity [378 ppm, 455. out of 13366, top 5%]	In HEK‐293FT and SH‐SY5Y cells (with T153/Y155 phospho‐ab in WB) [41]	Predicted to phase separate [79]
TK kinases				
Tyrosine‐protein kinase ABL1 (c‐ABL)	/	Cytoplasmic and nuclear expression at variable levels in all tissues with low tissue specificity [0.91 ppm, 6804. out of 13366]	Kinase assay with recombinant TDP‐43 (with radio‐labeled ATP in WB and with MS) [87] Directly interacts with TDP‐43 after IP from SH‐SY5Y cells [87]	/
AGC kinases				
cAMP‐dependent protein kinase catalytic subunit α and β (PKA, PRKACA and PRKACB)	/	Cytoplasmic localization, ubiquitous expression, high in skeletal muscle [113 ppm, 1172. out of 13366, top 10%]	Semi‐in vitro kinase assay with immuno‐purified proteins from HEK‐293FT cells (with S379, S403/S404 and S409/S410 phospho‐abs in WB) [88] In HEK‐293FT cells (with S379, S403/S404 and S409/S410 phospho‐abs in WB) [88] Directly interacts with TDP‐43 after IP from HEK‐293FT cells [88]	/

**TABLE 2:** Overview of proposed TDP‐43 phosphatases. Columns provide details on their links to TDP‐43 pathology, their subcellular localization and expression profile according to the Human Protein Atlas (www.proteinatlas.org) [70, 71], their protein abundance according to the PaxDb [72, 73] protein abundance database was integrated among multiple datasets in the brain [74, 75, 76, 77, 78], experimental evidence for their activity towards TDP‐43, and their links to phase separation and localization in membrane‐less organelles (MLOs) according to the RNA granule database [79].

TDP‐43 Phosphatases	Links to TDP‐43 pathology in AD/ALS/FTD patients	Localization/expression [protein abundance in the brain in parts per million (ppm) and abundance rank position out of all the identified proteins]	Experimental evidence for activity towards TDP‐43	Links to phase separation and MLOs
Protein phosphatase 3 catalytic subunit gamma (PPP3CC, Calcineurin)	Calcineurin co‑localizes with pTDP‑positive inclusions in FTLD‑TDP and ALS patient neurons [65]	Localized to the cytosol in addition localized to the nucleoplasm in most tissues with low tissue specificity [26.4 ppm, 2401. out of 13366, top 25%]	Identified through a Two‐hybrid screen in Yeast [65] in vitro dephosphorylation assay with recombinant proteins (with S409/S410 phospho‐ab in WB) [65] In *C. elegans* and in HEK293 cells (with S409/S410 phospho‐abs in WB and with S409/S410 phospho‐ab in IF) [65]	/
Protein Phosphatase 1 catalytic subunit alpha and gamma (PP1A and PP1G)	/	**Subunit alpha**: Localized to the nucleoplasm, plasma membrane, cytosol in several tissues with low tissue specificity [131 ppm, 1045. out of 13366, top 10%] **Subunit gamma**: Localized to the cytosol in several tissues with low tissue specificity [105 ppm, 1223. out of 13366, top 10%]	Identified from a candidate approach after an IP experiment [89] In HEK‐293FT cells (with S379, S403/S404 and S409/S410 phospho‐abs in WB) [89]	Found as stress granule (SG) interactor/CAPRIN1 interactors identified in sodium arsenite stressed HeLa cells [90]

**FIGURE 2:**
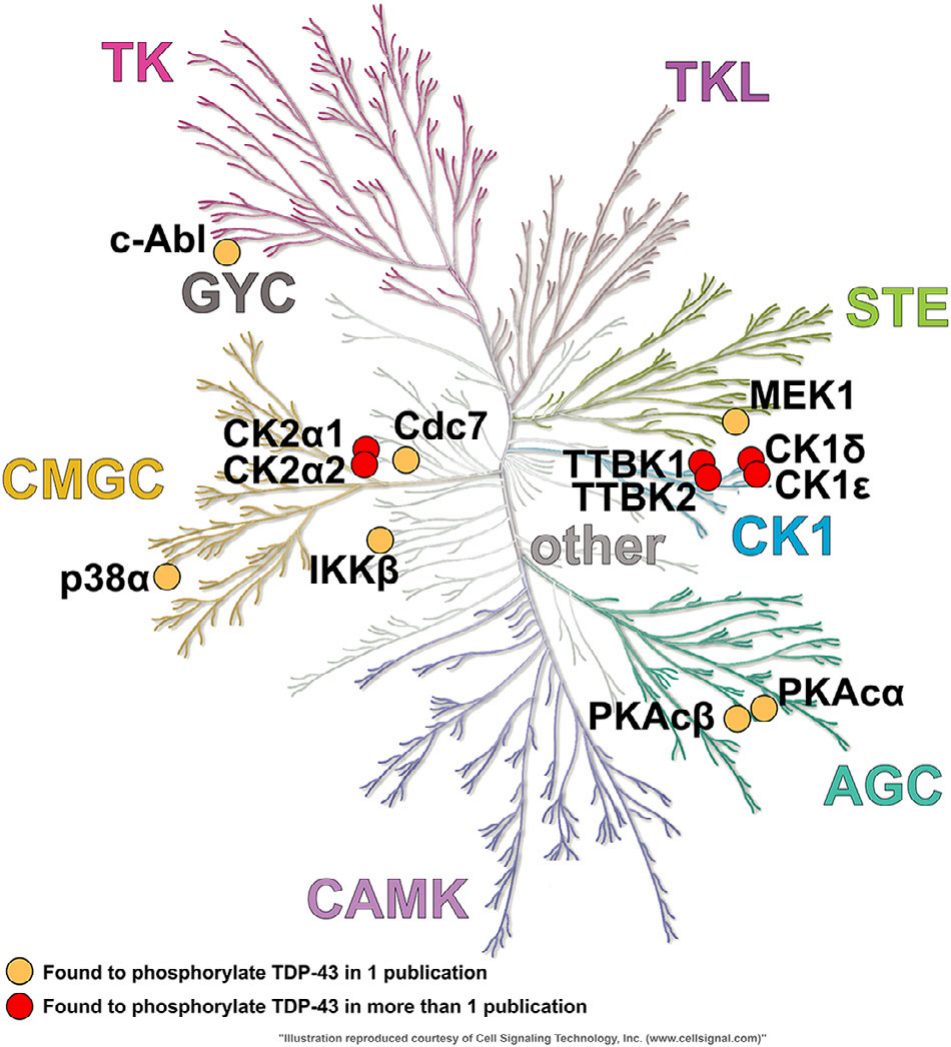
Kinase tree indicating the identified TDP‐43 kinases. Classification of kinase families based on sequence similarity and structural features. Kinase families represented are: Protein kinase A, G, and C families (AGC), Ca^2^⁺/calmodulin‐dependent kinases (CAMK), Casein kinase 1 family (CK1), CDK, MAPK, GSK, and CLK kinases (CMGC), Sterile 20‐like kinases (STE), Tyrosine kinases (TK), Tyrosine kinase‐like (TKL), Guanylate Cyclase kinases (GYC). Kinases reported to phosphorylate TDP‐43 in more than one publication are labeled with a red circle. Kinases reported to phosphorylate TDP‐43 in only one publication are labeled with an orange circle. The kinase tree vector image was obtained from KinMap (http://www.kinhub.org) [27] and adapted/reproduced with permission.

A major kinase family known to phosphorylate TDP‐43 is the CK1 family (Figure 2). In multiple different studies, CK1 kinases have been reported as potent regulators of TDP‐43 phosphorylation [51, 52], and they have been intensively studied as therapeutic targets in recent years [31, 45, 47, 48, 51, 52, 53, 54, 55, 56, 57, 58, 59]. Specifically, CK1δ was found to induce disease‐related phosphosites both in vitro and in cells [45, 47, 59]. Overexpression of CK1ε at both the transcript and protein level was found in cases of sporadic ALS and AD [55, 60, 61], and elevated levels of CK1δ, TTBK1 and TTBK2 were found in the affected brain regions of AD, FTD‐Tau, FTD‐TDP‐43, and ALS cases [56, 62, 63]. CK1δ and CK1ε were found to interact with TDP‐43 in pull‐down experiments [55, 59], and elevated CK1ε expression in AD brain correlated with high phospho‐S409/S410 TDP‐43 levels [55]. Additionally, TTBK1 and TTBK2 were found to co‐localize with phosphoS409/S410 TDP‐43 aggregates in spinal cord neurons from ALS patients [57].

Other kinase families remain more poorly characterized as potential TDP‐43 kinase, and most of them have been reported to phosphorylate TDP‐43 in only one publication (Figure 2, Table 1), hence, their relevance for TDP‐43 phosphorylation requires further validation.

To date, one comprehensive siRNA screen has aimed to identify kinases of S409/S410 TDP‐43 phosphorylation in *Caenorhabditis elegans*, leading to the identification of the worm orthologs of TTBK1, TTBK2, and Cdc7 [57, 64]. Similarly, a Yeast Two‐Hybrid screen identified Calcineurin as a phosphatase interacting with TDP‐43, which was subsequently validated in vitro and in cell models to dephosphorylate TDP‐43 [65]. More recently, Ko et al. compared the effects of seven different kinase inhibitors (specific for CK1δ, CK1ε, p38α/MAPK14, TTBK1, Cdc7, and CK2) on pS409/S410‐TDP‐43 levels and toxicity in SH‐SY5Y‐cells [52]. They concluded that CK1δ and CK1ε are the primary TDP‐43 kinases that affect TDP‐43 phosphorylation and aggregation in cellular models and thus should be considered as therapeutic targets.

Beyond these screens, no systems‐wide approaches or comprehensive kinase inhibitor screens have been performed so far to identify relevant TDP‐43 kinases or phosphatases. Given advancements in screening technologies and systems to model TDP‐43 pathology in human neurons [66, 67], a comprehensive screen to identify TDP‐43 kinases or phosphatases in a human pathology‐relevant system would be a desirable next step. Possible approaches could include CRISPR screens, large‐scale inhibitor screens and MS‐based approaches. For example, Kinase‐Catalyzed Crosslinking and Immunoprecipitation (K‐CLIP) followed by MS is emerging as a powerful tool for identifying novel kinase‐substrate interactions and would be well‐suited for this purpose [68]. Alternatively, BioID coupled with MS could be a method to identify kinases or phosphatases in proximity to TDP‐43, offering the advantage of detecting transient interactions that might be missed by traditional immunoprecipitation methods [69].

Identifying the kinases responsible for TDP‐43 phosphorylation in disease seems critical, as it might give us hints about the signaling pathways that are active in neuronal cells during disease progression. These kinases may not only act on TDP‐43, but also on many other proteins, that is, they may change the phospho‐proteome and thus change numerous interactions, structural conformations, activities and regulation of proteins. These molecular changes jointly could elicit, exacerbate or ameliorate disease progression.

Interestingly, some signaling pathways regulated by the most established TDP‐43 kinases (CK1 family kinases and CK2), such as the Wnt signaling pathway, circadian clock or FoxO signaling, were found to be misregulated in several neurodegenerative diseases, including ALS, AD, FTD, and HD [91, 92, 93, 94, 95, 96, 97, 98, 99, 100]. Thus, TDP‐43 hyperphosphorylation might be the result of a dysregulation in kinase signaling pathways in the brain, for example, caused by a faulty expression of certain kinases. Indeed, such a misregulated kinase expression has been reported for CK1ε, overexpressed both at the transcript and protein level in ALS and AD cases [55, 60, 61], and CK1δ, TTBK1, and TTBK2 at the protein level in AD, FTD and ALS cases [56, 62, 63]. Other kinases, such as Glycogen synthase kinase‐3 beta (GSK3β) and Protein kinase C alpha (PKCα) were found to be overexpressed in ALS and AD cases, respectively, and TANK binding kinase 1 (TBK1) and NIMA‐related kinase 1 (NEK1) loss‐of‐function mutations were identified in ALS patients, however these kinases have no direct link to TDP‐43 phosphorylation [61, 101, 102, 103]. Only a few studies have characterized the signaling networks in patient brains at the phospho‐proteome level [61], hence, we have little information on what downstream effects the misregulated or mutated kinases have on the proteome. Thus, more phospho‐proteome and proteome‐wide studies in diseased brains would be needed to better understand the cellular consequences of dysregulated kinase networks across different neurodegenerative diseases. This is not an easy task, as we are faced with the challenge that after death, ATP molecules are rapidly depleted, which immediately halts kinase activity, while phosphatase activity remains unaffected [104]. This introduces a significant bias and high variability between patients, as the remaining phospho‐sites strongly depend on the post‐mortem delay and protocols used for brain tissue preparation and conservation.

Overall, it can be speculated that hyperphosphorylation of TDP‐43 could reflect a general misregulation of kinases/phosphatases, that is, TDP‐43 hyperphosphorylation may just be the “tip of the iceberg” in the sea of a much wider misregulation of the (phospho)proteome.

Beyond the kinases that introduce the phospho‐sites on TDP‐43, another long‐standing question that researchers only recently started to address is: What are the consequences of altered TDP‐43 phosphorylation observed in disease? Does it contribute to TDP‐43 aggregation or TDP‐43 loss‐of‐function? Does it control TDP‐43's subcellular localization? Does it tune TDP‐43's interactions with other proteins or RNA/DNA molecules, or modulate its biophysical features, such as its ability to phase separate?

In the next paragraph, we will discuss what is known about the consequences of TDP‐43 hyperphosphorylation, in particular, how it affects TDP‐43's aggregation and phase separation behavior.

## 4 Effects of TDP‐43 Hyperphosphorylation on TDP‐43 Aggregation

**FIGURE 3:**
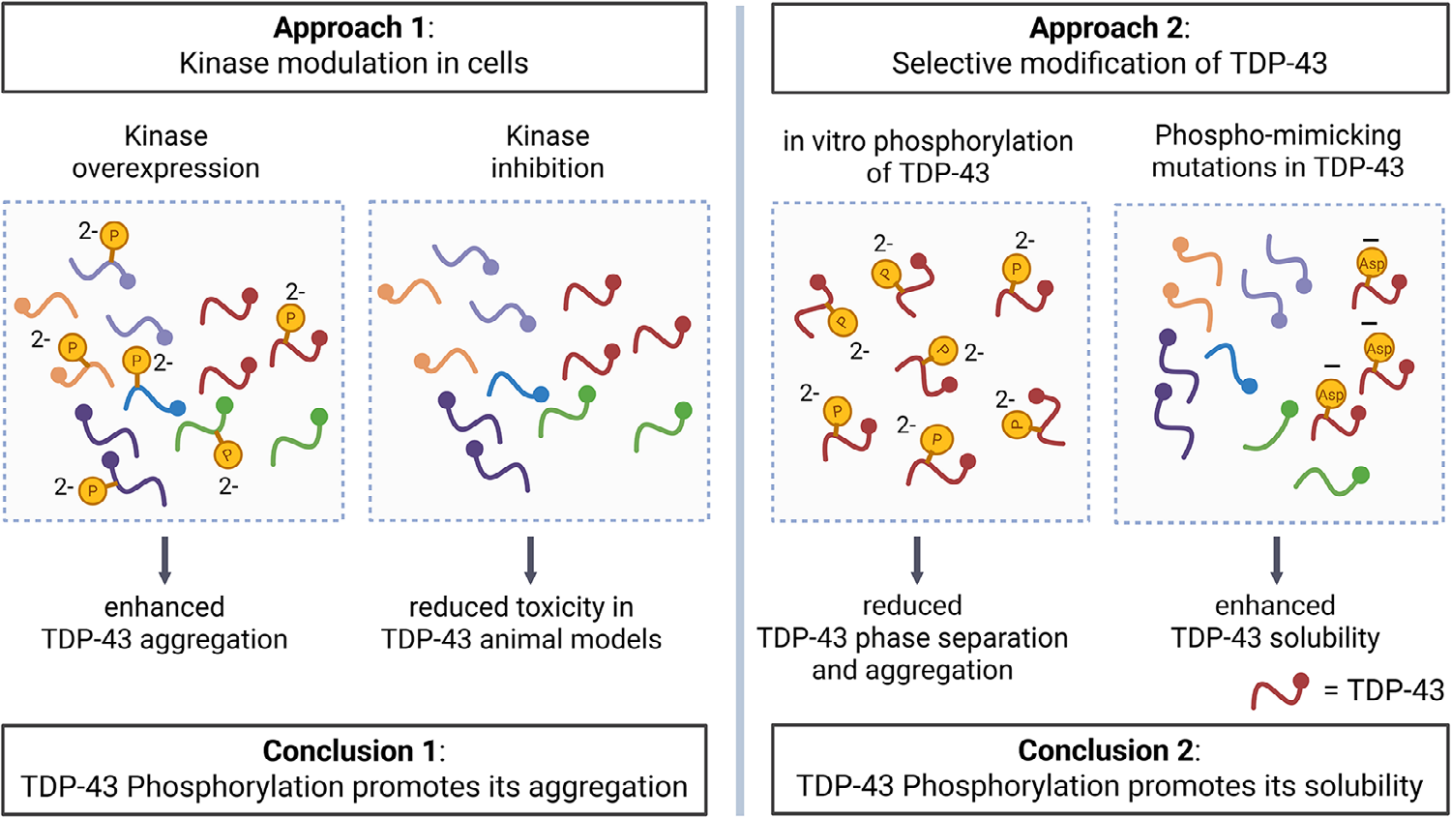
Scheme representing two alternative approaches used to study the effects of TDP‐43 phosphorylation on its aggregation behavior. For details, see the text. Created in BioRender. Dormann, D. (2025) https://BioRender.com/11b72n7.

Since TDP‐43 aggregates in post‐mortem brains of ALS/FTD patients appear to be hyperphosphorylated and consistently are positive for pS409/S410 by immunohistochemistry, it has been assumed early on that TDP‐43 phosphorylation might be a driver of its aggregation. Interestingly, there is experimental evidence for and against this hypothesis (Figure 3). The first approach was to overexpress or inhibit various TDP‐43 kinases in cell or animal models. This showed that overexpression of TDP‐43 kinases often promotes TDP‐43 aggregation and neurodegenerative phenotypes (Figure 3, left): for instance, overexpression of the kinase domain of CK1δ in SH‐SY5Y cells increases the amount of sarkosyl‐insoluble TDP‐43 and TDP‐43 aggregation in cultured cells [45]. Similarly, co‐expression of the CK1ε orthologue *Doubletime* in *drosophila* increases S409/S410 phosphorylation and TDP‐43 aggregation in flies [105]. Finally, TTBK1 (but not TTBK2) overexpression in *C. elegans* carrying a human TDP‐43 transgene causes a reduction in animal motility, that is, a neurodegenerative phenotype [62]. In line with these findings, pharmacologic inhibition of various TDP‐43 kinases was shown to have protective effects and to suppress TDP‐43 aggregation and toxicity (Figure 3, left): for example, pharmacological inhibition of TTBK1 rescues TDP‐43‐overexpression‐induced toxicity in iPSC‐derived neurons [56], and inhibition of p38α reduces TDP‐43 aggregation, cytoplasmic accumulation and toxicity in SH‐SY5Y cells [106]. Similarly, in *C. elegans*, a small molecule inhibitor of Cdc7 prevents TDP‐43–dependent neurodegeneration in TDP‐43–transgenic animals [64]. However, in all of these studies, the observed effects on TDP‐43 aggregation and/or toxicity cannot be unequivocally attributed to altered TDP‐43 phosphorylation, since kinases have numerous target proteins and regulate various cellular processes, such as signal transduction, metabolism and apoptosis. Thus, pharmacological inhibition of a specific kinase could well be beneficial, while overexpression of a kinase may be detrimental/toxic, however, these effects are not necessarily directly caused by changes to TDP‐43 phosphorylation, but may merely correlate with altered TDP‐43 phosphorylation. For making such a direct connection, one has to find ways to specifically alter phospho‐sites on TDP‐43, but no other phosphorylation events in the cell.

This has so far been attempted by phospho‐mimetic mutations (serine‐to‐aspartic acid or serine‐to‐glutamic acid exchanges) in the TDP‐43 sequence, which mimic the negative charges of phosphorylated serine/threonine residues (Figure 3, right). This approach demonstrated that introducing 2–12 phosphomimetic mutations in the C‐terminal region of TDP‐43 increases TDP‐43 solubility and decreases its aggregation propensity in *drosophila* as well as HEK293T cells [82, 107], and suppresses recruitment of TDP‐43 into stress‐induced condensates, such as stress granules, in HeLa cells and primary neurons [48, 108]. Additionally, using purified proteins, it was shown that increasing numbers of phospho‐mimetic residues in the TDP‐43 C‐terminus enhance the liquid‐like properties of TDP‐43 condensates and antagonize TDP‐43 aggregation in vitro [48]. Likewise, in vitro phosphorylation of TDP‐43 with CK1δ suppresses TDP‐43 phase separation and elevates its solubility [48] (Figure 3, right). Moreover, systems biology and computational simulations approaches furthermore support the hypothesis that TDP‐43 phosphorylation promotes its solubility: TDP‐43 was identified as one of the proteins that decreases in the soluble fraction and increases in the insoluble fraction upon phosphatase treatment in a proteome‐wide MS analysis [109]. Additionally, atomistic and coarse‐grained molecular dynamics simulations revealed that phosphomimetic and phosphorylated serines in the TDP‐43 LCR are more strongly solvated with water molecules and show reduced protein‐protein self‐interactions [48], and that phosphorylation of the TDP‐43 LCR with CK1δ causes dissolution of TDP‐43 LCR condensates [110].

One caveat of using phosphomimetic mutations is that they do not perfectly mimic phosphorylated serine or threonine residues, as they introduce only one negative charge, whereas a phosphate group carries two negative charges. Moreover, even if a certain phospho‐site on TDP‐43 is identified based on antibodies or MS, it does not mean that 100% of TDP‐43 molecules are phosphorylated in this position, as simulated by a phosphomimetic mutation. However, so far, we have a poor quantitative understanding of TDP‐43 phosphorylation in the disease state, as quantitative MS studies in larger patient cohorts are still missing.

An alternative approach could be to specifically recruit a TDP‐43 kinase using chemical inducers of proximity [111, 112]. An analogous approach has been recently successfully used to SUMOylate TDP‐43 in cells, by recruiting the SUMO ligase PIAS4 to TDP‐43 via the FKBP‐FKR dimerization system [113]. Applying such an approach to kinases in order to specifically hyperphosphorylate TDP‐43 could potentially allow to validate or disprove the above‐mentioned hypotheses on how TDP‐43 phosphorylation impacts its solubility, aggregation propensity and neurotoxicity.

Overall, considering the above‐mentioned caveat of the kinase overexpression/inhibition approach (Figure 3, left), it seems more likely that hyperphosphorylation promotes TDP‐43 solubility and antagonizes its aggregation (Figure 3, right), as this scenario is supported by a combination of in vitro, in cell, simulations and systems biology data. Accordingly, it has been proposed that TDP‐43 phosphorylation could be a response to cellular stress or to TDP‐43 insolubility [107, 114, 115] and could be a protective cellular mechanism to keep the protein in a soluble, dynamic and functional state [48]. In line with this model, it has recently been proposed that in FUS, another aggregation‐prone ALS/FTD‐linked RNA‐binding protein, phosphosites are more strongly enriched close to amyloidogenic protein regions, in order to prevent aberrant liquid‐to‐solid phase transitions of the protein in primates [116]. What remains to be addressed is how and by what trigger TDP‐43 phosphorylation is induced in cells. Another key question is whether kinases can still phosphorylate TDP‐43 in the condensed state, in amorphous aggregates or in the amyloid‐like state, and whether this can lead to the dissolution of TDP‐43 condensates or the amorphous or amyloid‐like aggregates observed in disease.

## 5 Effects of TDP‐43 Hyperphosphorylation on TDP‐43 Localization and RNA Regulatory Functions

A small number of studies reported how phospho‐mimetic mutations or kinase overexpression impact TDP‐43's nuclear localization, RNA binding and splicing activity. Multi‐site phosphomimetic substitution of 18 serine residues in the C‐terminal region (aa. 331–414, 18S→E) was shown to impair TDP‐43's splicing function, increase its nuclear retention and reduce mRNA binding and thus recruitment into stress granules under oxidative stress conditions [108]. Another study reported that phosphomimetic substitutions at residues S379, S403/404 or S409/410 enhance Tau exon 10 inclusion [117], whereas kinase overexpression (PKAcα, CK1δ, CK1ε) produced the opposite effect, suppressing Tau exon 10 inclusion [55, 59, 88]. In contrast, another study found that 12 phosphomimetic substitutions in the C‐terminal region (aa. 370–414, 12S→D) had no impact on RNA binding, autoregulation, splicing of several target RNAs or nuclear import of TDP‐43 [48]. These discrepancies may come from differences in the chosen phospho‐mimetic mutations or RNA targets analyzed. What is still missing is a systematic investigation of how phospho‐mimetic mutations alter TDP‐43's global RNA‐ and protein‐interactome as well as transcriptome‐wide alternative splicing or other DNA/RNA regulatory processes.

## 6 Potential Role of Condensates in TDP‐43 Phosphorylation

**FIGURE 4:**
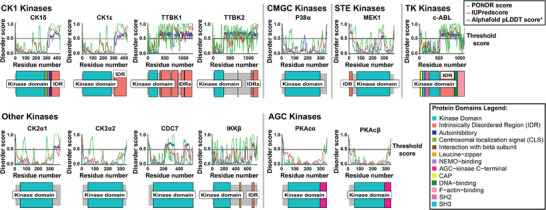
Disorder prediction scores for all putative TDP‐43 kinases grouped by kinase family. Scores are based on PONDR [123, 124], IUPred3 [125] and Alphafold2.0 [126, 127] databases and the information was retrieved in January 2025. A disorder prediction score above 0.5 indicates a disordered region. Protein domain cartoons are based on Uniprot annotation retrieved in January 2025 and visualized using the Bioconductor R package drawProteins [128]. *Alphafold pLDDT scores were recalculated with the formula new_score = 1 − (pLDDT score/100) for illustration purposes.

When looking over the list of potential TDP‐43 kinases (Table 1), it is notable that many of the candidate kinases localize to membrane‐less organelles (MLOs) that form by phase separation and have regions predicted to be intrinsically disordered (Figure 4). Thus, it can be speculated that TDP‐43 meets its kinases in condensates and that the pattern or rate of phosphorylation might be influenced by the condensate environment. Interestingly, it was recently shown that kinases and substrates that are co‐recruited into synthetic model condensates show an increase in phosphorylation efficiency by 3‐fold in vitro and by 100‐fold in vivo in budding yeast, while simultaneously showing decreased kinase specificity [118]. Tau was also phosphorylated ∼3‐fold more efficiently upon crowding‐induced phase separation in vitro, suggesting that the disease‐linked hyperphosphorylation pattern on Tau may stem from a combination of increased kinase efficiency and decreased kinase specificity in the condensate environment [118].

Whether TDP‐43 and its kinases indeed meet in cellular condensates, such as stress granules or other cytosolic or nuclear RNP granules, and to what extent the TDP‐43 phosphorylation rate and/or pattern in cells is influenced by the condensate environment remains to be addressed. Condensates may potentially influence kinase activity by concentrating and physically organizing enzymes and substrates and by providing distinct effective local environments compared to the surrounding solution [119, 120]. In support of this view, various nuclear condensates were recently shown to have different pH values [121, 122].

## 7 Open Questions Related to TDP‐43 Phosphorylation and Recommendations for Future Research

So far, research on TDP‐43 phoshorylation has been relatively unsystematic and a more comprehensive understanding of the mechanisms underlying TDP‐43 phosphorylation and its consequences is still required. This is surprising, given how relevant the topic is for many neurodegenerative diseases, including ALS, FTD, AD and LATE, and potentially even related degenerative conditions. One key question that urgently needs to be addressed is what stimuli/signals trigger TDP‐43 phosphorylation in cells and animal models, as this would allow us to model and further study this disease‐associated PTM in tractable model systems. Moreover, it will be interesting to study the role of condensates in TDP‐43 phosphorylation, that is, whether TDP‐43 phosphorylation is accelerated or suppressed in condensates and whether the condensates environment or their physicochemical properties influence TDP‐43 phosphorylation. Another key question is which kinases can phosphorylate TDP‐43 in the aggregated state, and whether this can lead to the dissolution of TDP‐43 aggregates and could be harnessed as a therapeutic strategy.

Additionally, we do not yet know if phosphorylation induces any conformational/structural changes to TDP‐43, as can be speculated from the fact that TDP‐43 has a neutrally charged IDR, for which multi‐site phosphorylation is expected to increase its radius of gyration, thus potentially creating a hub for multiple different interactors [129]. Along the same lines, it remains to be investigated how disease‐linked TDP‐43 phosphorylation influences its interaction with other proteins and/or nucleic acids. For example, it can be speculated that phosphorylation on TDP‐43 enhances interaction with the class of 14‐3‐3 proteins that bind to a precise phosphorylation consensus motif and were shown to bind to pathological TDP‐43 [130]. Other altered protein interactors could include E3 ligases, as phosphorylated TDP‐43 is consistently found to be polyubiquitinated in disease, raising the possibility that the two modifications could be linked.

In general, the interplay of TDP‐43 phosphorylation with other PTMs, such as methylation, SUMOylation, ubiquitinylation, citrullination and acetylation [131], is a highly relevant topic for future research. It has already been shown that monomethylation of R293 is antagonized by p38α‐mediated phosphorylation [86], and that SUMO2ylation at K408 directly competes with phosphorylation of residues S409/S410 [132]. However, this is just the tip of the iceberg, and much more cross‐regulation between PTMs is to be expected. Interesting PTMs for further investigations include SUMOylation and Ubiquitination, which mostly occur on multiple lysines in the NLS or RRM regions [133, 134]. Interestingly, SUMO2/3ylation is largely restricted to RNA‐free TDP‐43 and suppresses TDP‐43 aggregation, but also regulates its subcellular localization [133, 135, 136]. Another interesting PTM, which co‐occurs with phospho‐S409/S410 in patient tissues and mouse models, is citrullination within the RRMs or C‐terminal LCR [12, 137]. Citrullination removes arginine's positive charge and slows the amyloidogenic nucleation phase and reduces RNA‐binding of TDP‐43 [137]. Finally, acetylation is an interesting PTMs, as acetylation in the RRM regions compromises RNA binding and promotes the build‐up of phosphorylated and ubiquitinated TDP‐43 aggregates [138, 139, 140]. Together, these studies paint a picture of remarkable PTM complexity. The PTM landscape of TDP‐43 is likely highly dynamic, with modifications competing with or reinforcing one another, thus, a broad interplay among PTMs at overlapping or neighboring residues is to be expected. It would be highly desirable to gain a better understanding of this complex modification crosstalk and how it impacts TDP‐43 biology and pathology.

Answering these questions will be a fundamentally important step in further disentangling the role of TDP‐43 phosphorylation in disease and to finally clarify whether targeting this modification could be a promising disease‐modifying strategy.

## Author Contributions

S.M. and D.D. wrote and edited the manuscript. S.M. drafted and edited the figures and tables with input from D.D. D.D. acquired funding.

## Conflicts of Interest

The authors declare no conflicts of interest.

## Data Availability

Data sharing is not applicable to this article, as no datasets were generated or analyzed during the current study.

## References

[1] M. Jucker and L. C. Walker , “Propagation and Spread of Pathogenic Protein Assemblies in Neurodegenerative Diseases,” Nature Neuroscience 21, no. 10 (2018): 1341–1349, 10.1038/s41593-018-0238-6. 30258241 PMC6375686

[2] K. A. Josephs , J. L. Whitwell , S. D. Weigand , et al., “TDP‐43 Is a Key Player in the Clinical Features Associated With Alzheimer's Disease,” Acta Neuropathologica 127, no. 6 (2014): 811–824, 10.1007/s00401-014-1269-z. 24659241 PMC4172544

[3] S. Nag and J. A. Schneider , “Limbic‐Predominant Age‐Related TDP43 Encephalopathy (LATE) Neuropathological Change in Neurodegenerative Diseases,” Nature Reviews Neurology 19, no. 9 (2023): 525–541, 10.1038/s41582-023-00846-7. 37563264 PMC10964248

[4] M. Neumann , D. M. Sampathu , L. K. Kwong , et al., “Ubiquitinated TDP‐43 in Frontotemporal Lobar Degeneration and Amyotrophic Lateral Sclerosis,” Science (New York, NY) 314, no. 5796 (2006): 5796, 10.1126/science.1134108. 17023659

[5] R. T. Bjork , N. P. Mortimore , S. Loganathan , and D. C. Zarnescu , “Dysregulation of Translation in TDP‐43 Proteinopathies: Deficits in the RNA Supply Chain and Local Protein Production,” Frontiers in Neuroscience 16 (2022): 840357, 10.3389/fnins.2022.840357. 35321094 PMC8935057

[6] S.‐C. Ling , M. Polymenidou , and D. W. Cleveland , “Converging Mechanisms in ALS and FTD: Disrupted RNA and Protein Homeostasis,” Neuron 79, no. 3 (2013): 416–438, 10.1016/j.neuron.2013.07.033. 23931993 PMC4411085

[7] P. Tziortzouda , L. Van Den Bosch , and F. Hirth , “Triad of TDP43 Control in Neurodegeneration: Autoregulation, Localization and Aggregation,” Nature Reviews Neuroscience 22, no. 4 (2021): 197–208, 10.1038/s41583-021-00431-1. 33654312

[8] I. R. Mackenzie , R. Rademakers , and M. Neumann , “TDP‐43 and FUS in Amyotrophic Lateral Sclerosis and Frontotemporal Dementia,” Lancet Neurology 9, no. 10 (2010): 995–1007, 10.1016/S1474-4422(10)70195-2. 20864052

[9] J. R. Thorpe , H. Tang , J. Atherton , and N. J. Cairns , “Fine Structural Analysis of the Neuronal Inclusions of Frontotemporal Lobar Degeneration With TDP‐43 Proteinopathy,” Journal of Neural Transmission (Vienna, Austria: 1996) 115, no. 12 (2008): 1661–1671, 10.1007/s00702-008-0137-1. 18974920 PMC2789307

[10] A. Kerman , H.‐N. Liu , S. Croul , et al., “Amyotrophic Lateral Sclerosis Is a Non‐Amyloid Disease in Which Extensive Misfolding of SOD1 Is Unique to the Familial Form,” Acta Neuropathologica 119, no. 3 (2010): 335–344, 10.1007/s00401-010-0646-5. 20111867

[11] D. Arseni , T. Nonaka , M. H. Jacobsen , et al., “Heteromeric Amyloid Filaments of ANXA11 and TDP‐43 in FTLD‐TDP Type C,” Nature 634, no. 8034 (2024): 8034, 10.1038/s41586-024-08024-5. PMC11485244 39260416

[12] D. Arseni , M. Hasegawa , A. G. Murzin , et al., “Structure of Pathological TDP‐43 Filaments From ALS With FTLD,” Nature 601, no. 7891 (2022): 7891, 10.1038/s41586-021-04199-3. PMC7612255 34880495

[13] D. Arseni , R. Chen , A. G. Murzin , et al., “TDP‐43 Forms Amyloid Filaments With a Distinct Fold in Type A FTLD‐TDP,” Nature 620, no. 7975 (2023): 7975, 10.1038/s41586-023-06405-w. PMC10447236 37532939

[14] S. Alberti and D. Dormann , “Liquid–Liquid Phase Separation in Disease,” Annual Review of Genetics 53 (2019): 171–194, 10.1146/annurev-genet-112618-043527. 31430179

[15] H. B. Schmidt , A. Barreau , and R. Rohatgi , “Phase Separation‐Deficient TDP43 Remains Functional in Splicing,” Nature Communications 10, no. 1 (2019), 10.1038/s41467-019-12740-2. PMC6814767 31653829

[16] A. E. Conicella , G. H. Zerze , J. Mittal , and N. L. Fawzi , “ALS Mutations Disrupt Phase Separation Mediated by α‐Helical Structure in the TDP‐43 Low‐Complexity C‐Terminal Domain,” Structure (London, England: 1993) 24, no. 9 (2016): 9, 10.1016/j.str.2016.07.007. PMC5014597 27545621

[17] D. Dormann and E. A. Lemke , “Adding Intrinsically Disordered Proteins to Biological Ageing Clocks,” Nature Cell Biology 26, no. 6 (2024): 851–858, 10.1038/s41556-024-01423-w. 38783141

[18] C. M. Dobson , T. P. J. Knowles , and M. Vendruscolo , “The Amyloid Phenomenon and Its Significance in Biology and Medicine,” Cold Spring Harbor Perspectives in Biology 12, no. 2 (2020): a033878, 10.1101/cshperspect.a033878. 30936117 PMC6996456

[19] S. Alberti , “The Wisdom of Crowds: Regulating Cell Function Through Condensed States of Living Matter,” Journal of Cell Science 130, no. 17 (2017): 2789–2796, 10.1242/jcs.200295. 28808090

[20] A. E. Conicella , G. L. Dignon , G. H. Zerze , et al., “TDP‐43 α‐Helical Structure Tunes Liquid–Liquid Phase Separation and Function,” Proceedings of the National Academy of Sciences of the United States of America 117, no. 11 (2020): 5883–5894, 10.1073/pnas.1912055117. 32132204 PMC7084079

[21] S. Alberti and A. A. Hyman , “Biomolecular Condensates at the Nexus of Cellular Stress, Protein Aggregation Disease and Ageing,” Nature Reviews Molecular Cell Biology 22, no. 3 (2021): 196–213, 10.1038/s41580-020-00326-6. 33510441

[22] S. Alberti and A. A. Hyman , “Are Aberrant Phase Transitions a Driver of Cellular Aging?,” BioEssays: News and Reviews in Molecular, Cellular and Developmental Biology 38, no. 10 (2016): 959–968, 10.1002/bies.201600042. 27554449 PMC5108435

[23] M. Linsenmeier , L. Faltova , C. Morelli , et al., “The Interface of Condensates of the hnRNPA1 Low‐Complexity Domain Promotes Formation of Amyloid Fibrils,” Nature Chemistry 15, no. 10 (2023): 1340–1349, 10.1038/s41557-023-01289-9. PMC10533390 37749234

[24] Y. Shen , A. Chen , W. Wang , et al., “The Liquid‐to‐Solid Transition of FUS Is Promoted by the Condensate Surface,” Proceedings of the National Academy of Sciences of the United States of America 120, no. 33 (2023): 2301366120, 10.1073/pnas.2301366120. PMC10438845 37549257

[25] L. Emmanouilidis , E. Bartalucci , Y. Kan , et al., “A Solid Beta‐Sheet Structure Is Formed at the Surface of FUS Droplets During Aging,” Nature Chemical Biology 20, no. 8 (2024): 1044–1052, 10.1038/s41589-024-01573-w. 38467846 PMC11288893

[26] T. Das , F. K. Zaidi , M. Farag , et al., “Tunable Metastability of Condensates Reconciles Their Dual Roles in Amyloid Fibril Formation,” Molecular Cell 85, no. 11 (2025): 2230–2245.e7, 10.1016/j.molcel.2025.05.011. 40441157 PMC12831641

[27] J. Li , M. Zhang , W. Ma , et al., “Post‐Translational Modifications in Liquid‐Liquid Phase Separation: A Comprehensive Review,” Molecular Biomedicine 3, no. 1 (2022): 13, 10.1186/s43556-022-00075-2. 35543798 PMC9092326

[28] W. T. Snead and A. S. Gladfelter , “The Control Centers of Biomolecular Phase Separation: How Membrane Surfaces, PTMs, and Active Processes Regulate Condensation,” Molecular Cell 76, no. 2 (2019): 295–305, 10.1016/j.molcel.2019.09.016. 31604601 PMC7173186

[29] M. Hofweber and D. Dormann , “Friend or Foe—Post‐Translational Modifications as Regulators of Phase Separation and RNP Granule Dynamics,” Journal of Biological Chemistry 294, no. 18 (2019): 7137–7150, 10.1074/jbc.TM118.001189. 30587571 PMC6509508

[30] M. Neumann , L. K. Kwong , E. B. Lee , et al., “Phosphorylation of S409/410 of TDP‐43 Is a Consistent Feature in all Sporadic and Familial Forms of TDP‐43 Proteinopathies,” Acta Neuropathologica 117, no. 2 (2009): 137–149, 10.1007/s00401-008-0477-9. 19125255 PMC2693625

[31] M. Hasegawa , T. Arai , T. Nonaka , et al., “Phosphorylated TDP‐43 in Frontotemporal Lobar Degeneration and Amyotrophic Lateral Sclerosis,” Annals of Neurology 64, no. 1 (2008): 60–70, 10.1002/ana.21425. 18546284 PMC2674108

[32] M. Neumann , P. Frick , F. Paron , J. Kosten , E. Buratti , and I. R. Mackenzie , “Antibody Against TDP‐43 Phosphorylated At Serine 375 Suggests Conformational Differences of TDP‐43 Aggregates Among FTLD–TDP Subtypes,” Acta Neuropathologica 140, no. 5 (2020): 645–658, 10.1007/s00401-020-02207-w. 32778941 PMC7547034

[33] J. I. López‐Carbonero , I. García‐Toledo , L. Fernández‐Hernández , et al., “In Vivo Diagnosis of TDP‐43 Proteinopathies: In Search of Biomarkers of Clinical Use,” Translational Neurodegeneration 13, no. 1 (2024): 29, 10.1186/s40035-024-00419-8. 38831349 PMC11149336

[34] T. Arai , I. R. A. Mackenzie , M. Hasegawa , et al., “Phosphorylated TDP‐43 in Alzheimer's Disease and Dementia With Lewy Bodies,” Acta Neuropathologica 117, no. 2 (2009): 125–136, 10.1007/s00401-008-0480-1. 19139911

[35] P. Smethurst , E. Risse , G. E. Tyzack , et al., “Distinct Responses of Neurons and Astrocytes to TDP‐43 Proteinopathy in Amyotrophic Lateral sclerosis,” Brain: A Journal of Neurology 143, no. 2 (2020): 430–440, 10.1093/brain/awz419. 32040555 PMC7009461

[36] P. T. Nelson , D. W. Dickson , J. Q. Trojanowski , et al., “Limbic‐Predominant Age‐Related TDP‐43 Encephalopathy (LATE): Consensus Working Group Report,” Brain: A Journal of Neurology 142, no. 6 (2019): 1503–1527, 10.1093/brain/awz099. 31039256 PMC6536849

[37] E. Gelpi , I. Aldecoa , D. Lopez‐Villegas , et al., “Atypical Astroglial pTDP‐43 Pathology in Astroglial Predominant Tauopathy,” Neuropathology and Applied Neurobiology 47, no. 7 (2021): 1109–1113, 10.1111/nan.12707. 33730418 PMC9292602

[38] J. Santiago , D. Pocevičiūtė , N. B. Bank , and M. Wennström , “Perivascular Phosphorylated TDP ‐43 Inclusions Are Associated With Alzheimer's Disease Pathology and Loss of CD146 and Aquaporin‐4,” Brain Pathology (Zurich, Switzerland) 35, no. 2 (2025): 13304, 10.1111/bpa.13304. PMC11835440 39251230

[39] L. Cracco , E. H. Doud , G. I. Hallinan , et al., “Distinguishing Post‐Translational Modifications in dominantly Inherited Frontotemporal Dementias: FTLD‐TDP Type A (GRN) vs. Type B (C9orf72),” Neuropathology and Applied Neurobiology 48, no. 6 (2022): 12836, 10.1111/nan.12836. PMC9452479 35836354

[40] F. Kametani , T. Obi , T. Shishido , et al., “Mass Spectrometric Analysis of Accumulated TDP‐43 in Amyotrophic Lateral Sclerosis Brains,” Scientific Reports 6 (2016): 23281, 10.1038/srep23281. 26980269 PMC4793195

[41] W. Li , A. N. Reeb , B. Lin , et al., “Heat Shock‐Induced Phosphorylation of TAR DNA‐Binding Protein 43 (TDP‐43) by MAPK/ERK Kinase Regulates TDP‐43 Function,” Journal of Biological Chemistry 292, no. 12 (2017): 5089–5100, 10.1074/jbc.M116.753913. 28167528 PMC5377819

[42] J. V. Olsen , B. Blagoev , F. Gnad , et al., “Global, In Vivo, and Site‐Specific Phosphorylation Dynamics in Signaling Networks,” Cell 127, no. 3 (2006): 635–648, 10.1016/j.cell.2006.09.026. 17081983

[43] P. Mertins , D. R. Mani , K. V. Ruggles , et al., “Proteogenomics Connects Somatic Mutations to Signalling in Breast Cancer,” Nature 534, no. 7605 (2016): 55–62, 10.1038/nature18003. 27251275 PMC5102256

[44] S. A. Stuart , S. Houel , T. Lee , N. Wang , W. M. Old , and N. G. Ahn , “A Phosphoproteomic Comparison of B‐RAFV600E and MKK1/2 Inhibitors in Melanoma Cells*,” Molecular & Cellular Proteomics: MCP 14, no. 6 (2015): 1599–1615, 10.1074/mcp.M114.047233. 25850435 PMC4458723

[45] T. Nonaka , G. Suzuki , Y. Tanaka , et al., “Phosphorylation of TAR DNA‐Binding Protein of 43 kDa (TDP‐43) by Truncated Casein Kinase 1δ Triggers Mislocalization and Accumulation of TDP‐43,” Journal of Biological Chemistry 291, no. 11 (2016): 5473–5483, 10.1074/jbc.M115.695379. 26769969 PMC4786690

[46] K. T. G. Rigbolt , T. A. Prokhorova , V. Akimov , et al., “System‐Wide Temporal Characterization of the Proteome and Phosphoproteome of Human Embryonic Stem Cell Differentiation,” Science Signaling 4, no. 164 (2011): rs3, 10.1126/scisignal.2001570. 21406692

[47] F. Kametani , T. Nonaka , T. Suzuki , et al., “Identification of Casein Kinase‐1 Phosphorylation Sites on TDP‐43,” Biochemical and Biophysical Research Communications 382, no. 2 (2009): 405–409, 10.1016/j.bbrc.2009.03.038. 19285963

[48] L. A. Gruijs da Silva , F. Simonetti , S. Hutten , et al., “Disease‐Linked TDP‐43 Hyperphosphorylation Suppresses TDP‐43 Condensation and Aggregation,” EMBO Journal 41, no. 8 (2022): 108443, 10.15252/embj.2021108443. PMC9016352 35112738

[49] P. V. Hornbeck , B. Zhang , B. Murray , J. M. Kornhauser , V. Latham , and E. Skrzypek , “PhosphoSitePlus, 2014: Mutations, PTMs and Recalibrations,” Nucleic Acids Research 43, no. Database issue (2015): D512–520, 10.1093/nar/gku1267. 25514926 PMC4383998

[50] T. Arakhamia , C. E. Lee , Y. Carlomagno , et al., “Post‐Translational Modifications Mediate the Structural Diversity of Tauopathy Strains,” Cell 180, no. 4 (2020): 633–644.e12, 10.1016/j.cell.2020.01.027. 32032505 PMC7491959

[51] V. I. Ko , K. Ong , D. Y. Kwon , et al., “CK1δ/ε‐Mediated TDP‐43 Phosphorylation Contributes to Early Motor Neuron Disease Toxicity in Amyotrophic Lateral sclerosis,” Acta Neuropathologica Communications 12, no. 1 (2024), 10.1186/s40478-024-01902-z. PMC11619411 39633494

[52] V. I. Ko , K. Ong , D. W. Cleveland , H. Yu , and J. M. Ravits , “CK1δ/ε Kinases Regulate TDP‐43 Phosphorylation and Are Therapeutic Targets for ALS‐Related TDP‐43 Hyperphosphorylation,” Neurobiology of Disease 196 (2024): 106516, 10.1016/j.nbd.2024.106516. 38677657

[53] V. Nozal , L. Martínez‐González , M. Gomez‐Almeria , et al., “TDP‐43 Modulation by Tau‐Tubulin Kinase 1 Inhibitors: A New Avenue for Future Amyotrophic Lateral Sclerosis Therapy,” Journal of Medicinal Chemistry 65, no. 2 (2022): 1585–1607, 10.1021/acs.jmedchem.1c01942. 34978799

[54] L. Martinez‐Gonzalez , E. P. Cuevas , C. Tosat‐Bitrián , et al., “TTBK1 and CK1 Inhibitors Restore TDP‐43 Pathology and Avoid Disease Propagation in Lymphoblast From Alzheimer's Disease Patients,” Frontiers in Molecular Neuroscience 16 (2023): 1243277, 10.3389/fnmol.2023.1243277. 37621404 PMC10445132

[55] J. Gu , W. Hu , X. Tan , et al., “Elevation of Casein Kinase 1ε Associated With TDP‐43 and Tau Pathologies in Alzheimer's Disease,” Brain Pathology (Zurich, Switzerland) 30, no. 2 (2020): 283–297, 10.1111/bpa.12775. 31376192 PMC8018014

[56] Y. Tian , Y. Wang , A. M. Jablonski , et al., “Tau‐Tubulin Kinase 1 Phosphorylates TDP‐43 at Disease‐Relevant Sites and Exacerbates TDP‐43 Pathology,” Neurobiology of Disease 161 (2021): 105548, 10.1016/j.nbd.2021.105548. 34752923

[57] N. F. Liachko , P. J. McMillan , T. J. Strovas , et al., “The Tau Tubulin Kinases TTBK1/2 Promote Accumulation of Pathological TDP‐43,” PLoS Genetics 10, no. 12 (2014): 1004803, 10.1371/journal.pgen.1004803. PMC4256087 25473830

[58] F. M. Bashore , A. B. Marquez , A. Chaikuad , et al., “Modulation of Tau Tubulin Kinases (TTBK1 and TTBK2) Impacts Ciliogenesis,” Scientific Reports 13, no. 1 (2023): 6118, 10.1038/s41598-023-32854-4. 37059819 PMC10104807

[59] M. Yang , R. Qi , Y. Liu , et al., “Casein Kinase 1δ Phosphorylates TDP‐43 and Suppresses Its Function in Tau mRNA Processing,” Journal of Alzheimer's Disease: JAD 91, no. 4 (2023): 1527–1539, 10.3233/JAD-220985. 36641675

[60] F. Krach , R. Batra , E. C. Wheeler , et al., “Transcriptome–Pathology Correlation Identifies Interplay Between TDP‐43 and the Expression of its Kinase CK1E in Sporadic ALS,” Acta Neuropathologica 136, no. 3 (2018): 405–423, 10.1007/s00401-018-1870-7. 29881994 PMC6215775

[61] N. Morshed , M. J. Lee , F. H. Rodriguez , D. A. Lauffenburger , D. Mastroeni , and F. M. White , “Quantitative Phosphoproteomics Uncovers Dysregulated Kinase Networks in Alzheimer's Disease,” Nature Aging 1, no. 6 (2021): 550–565, 10.1038/s43587-021-00071-1. 37117831

[62] L. M. Taylor , P. J. McMillan , N. F. Liachko , et al., “Pathological Phosphorylation of Tau and TDP‐43 by TTBK1 and TTBK2 Drives Neurodegeneration,” Molecular Neurodegeneration 13, no. 1 (2018): 7, 10.1186/s13024-018-0237-9. 29409526 PMC5802059

[63] N. Ghoshal , J. F. Smiley , A. J. DeMaggio , et al., “A New Molecular Link Between the Fibrillar and Granulovacuolar Lesions of Alzheimer's Disease,” American Journal of Pathology 155, no. 4 (1999): 1163–1172, 10.1016/S0002-9440(10)65219-4. 10514399 PMC1867028

[64] N. F. Liachko , P. J. McMillan , C. R. Guthrie , T. D. Bird , J. B. Leverenz , and B. C. Kraemer , “CDC7 Inhibition Blocks Pathological TDP‐43 Phosphorylation and Neurodegeneration,” Annals of Neurology 74, no. 1 (2013): 39–52, 10.1002/ana.23870. 23424178 PMC3775949

[65] N. F. Liachko , A. D. Saxton , P. J. McMillan , et al., “The Phosphatase Calcineurin Regulates Pathological TDP‐43 Phosphorylation,” Acta Neuropathologica 132, no. 4 (2016): 545–561, 10.1007/s00401-016-1600-y. 27473149 PMC5026939

[66] C. Scialò , W. Zhong , S. Jagannath , et al., “Seeded Aggregation of TDP‐43 Induces Its Loss of Function and Reveals Early Pathological Signatures,” Neuron 113, no. 10 (2025): 1614–1628.e11, 10.1016/j.neuron.2025.03.008. 40157355

[67] J. Rummens , B. Khalil , G. Yıldırım , et al., “TDP‐43 Seeding Induces Cytoplasmic Aggregation Heterogeneity and Nuclear Loss of Function of TDP‐43,” Neuron 113, no. 10 (2025): 1597–1613.e8, 10.1016/j.neuron.2025.03.004. 40157356

[68] R. J. Beltman and M. K. H. Pflum , “Kinase‐Catalyzed Crosslinking and Immunoprecipitation (K‐CLIP) to Explore Kinase‐Substrate Pairs,” Current Protocols 2, no. 9 (2022): 539, 10.1002/cpz1.539. PMC9885979 36135312

[69] R. M. Sears , D. G. May , and K. J. Roux , “BioID as a Tool for Protein‐Proximity Labeling in Living Cells,” Methods in Molecular Biology (Clifton, NJ), 2012 (2019): 299–313, 10.1007/978-1-4939-9546-2_15. PMC6583792 31161514

[70] P. J. Thul , L. Åkesson , M. Wiking , et al., “A Subcellular Map of the Human Proteome,” Science (New York, NY) 356, no. 6340 (2017): aal3321, 10.1126/science.aal3321. 28495876

[71] M. Uhlén , L. Fagerberg , B. M. Hallström , et al., “Proteomics. Tissue‐Based Map of the Human Proteome,” Science (New York, NY) 347, no. 6220 (2015): 1260419, 10.1126/science.1260419. 25613900

[72] M. Wang , C. J. Herrmann , M. Simonovic , D. Szklarczyk , and C. von Mering , “Version 4.0 of PaxDb: Protein abundance data, integrated Across model organisms, tissues, and cell‐lines,” Proteomics 15, no. 18 (2015): 163–168, 10.1002/pmic.201400441. PMC6680238 25656970

[73] Q. Huang , D. Szklarczyk , M. Wang , M. Simonovic , and C. von Mering , “PaxDb 5.0: Curated Protein Quantification Data Suggests Adaptive Proteome Changes in Yeasts,” Molecular & Cellular Proteomics: MCP 22, no. 10 (2023): 100640, 10.1016/j.mcpro.2023.100640. 37659604 PMC10551891

[74] L. Martens , M. Müller , C. Stephan , et al., “A Comparison of the HUPO Brain Proteome Project Pilot With Other Proteomics Studies,” Proteomics 6, no. 18 (2006): 5076–5086, 10.1002/pmic.200600291. 16912975

[75] E. W. Deutsch , Z. Sun , D. Campbell , et al., “State of the Human Proteome in 2014/2015 As Viewed Through PeptideAtlas: Enhancing Accuracy and Coverage Through the AtlasProphet,” Journal of Proteome Research 14, no. 9 (2015): 3461–3473, 10.1021/acs.jproteome.5b00500. 26139527 PMC4755269

[76] M. S. Kim , S. M. Pinto , D. Getnet , et al., “A Draft Map of the Human Proteome,” *Nature* no. 7502 (2014): 509, 10.1038/nature13302. PMC4403737 24870542

[77] D. Wang , B. Eraslan , T. Wieland , et al., “A Deep Proteome and Transcriptome Abundance Atlas of 29 Healthy Human Tissues,” Molecular Systems Biology 15, no. 2 (2019): 8503, 10.15252/msb.20188503. PMC6379049 30777892

[78] T. Farrah , E. W. Deutsch , M. R. Hoopmann , et al., “The State of the Human Proteome in 2012 as Viewed Through PeptideAtlas,” Journal of Proteome Research 12, no. 1 (2013): 162–171, 10.1021/pr301012j. 23215161 PMC3928036

[79] S. R. Millar , J. Q. Huang , K. J. Schreiber , et al., “A New Phase of Networking: The Molecular Composition and Regulatory Dynamics of Mammalian Stress Granules,” Chemical Reviews 123, no. 14 (2023): 9036–9064, 10.1021/acs.chemrev.2c00608. 36662637 PMC10375481

[80] B. Zhang , Q. Shi , S. N. Varia , et al., “The Activity‐Dependent Regulation of Protein Kinase Stability by the Localization to P‐Bodies,” Genetics 203, no. 3 (2016): 1191–1202, 10.1534/genetics.116.187419. 27182950 PMC4937477

[81] Y. Carlomagno , Y. Zhang , M. Davis , et al., “Casein Kinase II Induced Polymerization of Soluble TDP‐43 Into Filaments Is Inhibited by Heat Shock Proteins,” PLoS ONE 9, no. 3 (2014): 90452, 10.1371/journal.pone.0090452. PMC3942448 24595055

[82] H.‐Y. Li , P.‐A. Yeh , H.‐C. Chiu , C.‐Y. Tang , and B. P. Tu , “Hyperphosphorylation as a Defense Mechanism to Reduce TDP‐43 Aggregation,” PLoS ONE 6, no. 8 (2011): 23075, 10.1371/journal.pone.0023075. PMC3151276 21850253

[83] K.‐H. Lee , P. Zhang , H. J. Kim , et al., “C9orf72 Dipeptide Repeats Impair the Assembly, Dynamics, and Function of Membrane‐Less Organelles,” Cell 167, no. 3 (2016): 774–788.e17, 10.1016/j.cell.2016.10.002. 27768896 PMC5079111

[84] D. Berchtold , N. Battich , and L. Pelkmans , “A Systems‐Level Study Reveals Regulators of Membrane‐Less Organelles in Human Cells,” Molecular Cell 72, no. 6 (2018): 1035–1049.e5, 10.1016/j.molcel.2018.10.036. 30503769

[85] Y. Iguchi , Y. Takahashi , J. Li , et al., “IκB Kinase Phosphorylates Cytoplasmic TDP‐43 and Promotes Its Proteasome Degradation,” Journal of Cell Biology 223, no. 2 (2024): 202302048, 10.1083/jcb.202302048. PMC10783433 38197897

[86] M. Aikio , H. M. Odeh , H. J. Wobst , et al., “Opposing Roles of p38α‐Mediated Phosphorylation and PRMT1‐Mediated Arginine Methylation in Driving TDP‐43 Proteinopathy,” Cell Reports 44, no. 1 (2025): 115205, 10.1016/j.celrep.2024.115205. 39817908 PMC11831926

[87] S. Lee , H. G. Ryu , S. H. Kweon , et al., “C‐Abl Regulates the Pathological Deposition of TDP‐43 via Tyrosine 43 Phosphorylation,” Cells 11, no. 24 (2022): 3972, 10.3390/cells11243972. 36552734 PMC9776721

[88] J. Gu , D. Chu , N. Jin , F. Chen , and F. Liu , “Cyclic AMP‐Dependent Protein Kinase Phosphorylates TDP‐43 and Modulates Its Function in Tau mRNA Processing,” Journal of Alzheimer's Disease: JAD 70, no. 4 (2019): 1093–1102, 10.3233/JAD-190368. 31306131

[89] J. Gu , W. Wang , S. Miao , et al., “Protein Phosphatase 1 Dephosphorylates TDP ‐43 and Suppresses Its Function in Tau Exon 10 Inclusion,” FEBS Letters 592, no. 3 (2018): 402–410, 10.1002/1873-3468.12976. 29334120

[90] L. Vu , A. Ghosh , C. Tran , et al., “Defining the Caprin‐1 Interactome in Unstressed and Stressed Conditions,” Journal of Proteome Research 20, no. 6 (2021): 3165–3178, 10.1021/acs.jproteome.1c00016. 33939924 PMC9083243

[91] L. Liu , J. Bai , F. Liu , et al., “Cross‐Talking Pathways of Forkhead Box O1 (FOXO1) Are Involved in the Pathogenesis of Alzheimer's Disease and Huntington's Disease,” Oxidative Medicine and Cellular Longevity 2022 (2022): 7619255, 10.1155/2022/7619255. 35154571 PMC8831070

[92] C. Pinto , D. B. Medinas , F. Fuentes‐Villalobos , et al., “β‐Catenin Aggregation in Models of ALS Motor Neurons: GSK3β Inhibition Effect and Neuronal Differentiation,” Neurobiology of Disease 130 (2019): 104497, 10.1016/j.nbd.2019.104497. 31176720

[93] C. Pinto , P. Cárdenas , N. Osses , and J. P. Henríquez , “Characterization of Wnt/β‐catenin and BMP/Smad Signaling Pathways in an In Vitro Model of Amyotrophic Lateral Sclerosis,” Frontiers in Cellular Neuroscience 7 (2013): 239, 10.3389/fncel.2013.00239. 24348333 PMC3847560

[94] X. Jiang , Y. Guan , Z. Zhao , et al., “Potential Roles of the WNT Signaling Pathway in Amyotrophic Lateral Sclerosis,” Cells 10, no. 4 (2021): 839, 10.3390/cells10040839. 33917816 PMC8068170

[95] C. Elliott , A. I. Rojo , E. Ribe , et al., “A Role for APP in Wnt Signalling Links Synapse Loss With β‐Amyloid Production,” Translational Psychiatry 8, no. 1 (2018): 179, 10.1038/s41398-018-0231-6. 30232325 PMC6145937

[96] Z. Huang , Q. Liu , Y. Peng , et al., “Circadian Rhythm Dysfunction Accelerates Disease Progression in a Mouse Model With Amyotrophic Lateral Sclerosis,” Frontiers in Neurology 9 (2018): 218, 10.3389/fneur.2018.00218. 29740382 PMC5928145

[97] M. G. Figueiro , “Light, Sleep and Circadian Rhythms in Older Adults With Alzheimer's Disease and Related Dementias,” Neurodegenerative Disease Management 7, no. 2 (2017): 119–145, 10.2217/nmt-2016-0060. 28534696 PMC5836917

[98] L. Rigat , K. Ouk , A. Kramer , and J. Priller , “Dysfunction of Circadian and Sleep Rhythms in the Early Stages of Alzheimer's Disease,” Acta Physiologica (Oxford, England) 238, no. 2 (2023): 13970, 10.1111/apha.13970. 37000425

[99] K. N. Anderson , C. Hatfield , C. Kipps , M. Hastings , and J. R. Hodges , “Disrupted Sleep and Circadian Patterns in Frontotemporal Dementia,” European Journal of Neurology 16, no. 3 (2009): 317–323, 10.1111/j.1468-1331.2008.02414.x. 19170747

[100] N. Cermakian , E. W. Lamont , P. Boudreau , and D. B. Boivin , “Circadian Clock Gene Expression in Brain Regions of Alzheimer's disease Patients and Control Subjects,” Journal of Biological Rhythms 26, no. 2 (2011): 160–170, 10.1177/0748730410395732. 21454296

[101] W. Yang , C. Leystra‐Lantz , and M. J. Strong , “Upregulation of GSK3β Expression in Frontal and Temporal Cortex in ALS With Cognitive Impairment (ALSci),” Brain Research 1196 (2008): 131–139, 10.1016/j.brainres.2007.12.031. 18221734

[102] S. I. Alfonso , J. A. Callender , B. Hooli , et al., “Gain‐of‐Function Mutations in Protein Kinase Cα (PKCα) May Promote Synaptic Defects in Alzheimer's Disease,” Science Signaling 9, no. 427 (2016): ra47, 10.1126/scisignal.aaf6209. 27165780 PMC5154619

[103] G. Lordén , J. M. Wozniak , K. Doré , et al., “Enhanced Activity of Alzheimer Disease‐Associated Variant of Protein Kinase Cα Drives Cognitive Decline in a Mouse Model,” Nature Communications 13, no. 1 (2022): 7200, 10.1038/s41467-022-34679-7. PMC9684486 36418293

[104] Y. Wang , Y. Zhang , W. Hu , et al., “Rapid Alteration of Protein Phosphorylation During Postmortem: Implication in the Study of Protein Phosphorylation,” Scientific Reports 5 (2015): 15709, 10.1038/srep15709. 26511732 PMC4625177

[105] D. K. Choksi , B. Roy , S. Chatterjee , et al., “TDP‐43 Phosphorylation by Casein Kinase Iε Promotes Oligomerization and Enhances Toxicity In Vivo,” Human Molecular Genetics 23, no. 4 (2014): 1025–1035, 10.1093/hmg/ddt498. 24105464

[106] M. Aikio , H. J. Wobst , H. M. Odeh , et al., “Opposing Roles of p38α‐mediated Phosphorylation and Arginine Methylation in Driving TDP‐43 Proteinopathy (p. 2021.08.04.455154),” BioRxiv (2021), 10.1101/2021.08.04.455154.

[107] O. A. Brady , P. Meng , Y. Zheng , Y. Mao , and F. Hu , “Regulation of TDP‐43 Aggregation by Phosphorylation and p62/SQSTM1,” Journal of Neurochemistry 116, no. 2 (2011): 248–259, 10.1111/j.1471-4159.2010.07098.x. 21062285

[108] C. Rabhi , N. Babault , C. Martin , et al., “TDP‐43 Nuclear Retention Is Antagonized by Hypo‐Phosphorylation of Its C‐Terminus in the Cytoplasm,” Communications Biology 8, no. 1 (2025): 136, 10.1038/s42003-025-07456-7. 39875548 PMC11775348

[109] S. R. Kundinger , E. B. Dammer , L. Yin , et al., “Phosphorylation Regulates Arginine‐rich RNA‐Binding Protein Solubility and Oligomerization,” Journal of Biological Chemistry 297, no. 5 (2021): 101306, 10.1016/j.jbc.2021.101306. 34673031 PMC8569591

[110] E. Zippo , D. Dormann , T. Speck , and L. S. Stelzl , “Molecular Simulations of Enzymatic Phosphorylation of Disordered Proteins and Their Condensates,” Nature Communications 16, no. 1 (2025): 4649, 10.1038/s41467-025-59676-4. PMC12089381 40389455

[111] M. Konstantinidou and M. R. Arkin , “Molecular Glues for Protein‐Protein Interactions: Progressing Toward a New Dream,” Cell Chemical Biology 31, no. 6 (2024): 1064–1088, 10.1016/j.chembiol.2024.04.002. 38701786 PMC11193649

[112] S. U. Siriwardena , D. N. P. Munkanatta Godage , V. M. Shoba , et al., “Phosphorylation‐Inducing Chimeric Small Molecules,” Journal of the American Chemical Society 142, no. 33 (2020): 14052–14057, 10.1021/jacs.0c05537. 32787262

[113] K. Wagner , J. Keiten‐Schmitz , B. Adhikari , et al., “Induced Proximity to PML Protects TDP‐43 From Aggregation via SUMO – Ubiquitin Networks,” Nature Chemical Biology (2025), 10.1038/s41589-025-01886-4. PMC12394070 40246979

[114] D. Dormann , A. Capell , A. M. Carlson , et al., “Proteolytic Processing of TAR DNA Binding Protein‐43 by Caspases Produces C‐Terminal Fragments With Disease Defining Properties Independent of Progranulin,” Journal of Neurochemistry 110, no. 3 (2009): 1082–1094, 10.1111/j.1471-4159.2009.06211.x. 19522733

[115] P. Zhang , B. Fan , P. Yang , et al., “Chronic Optogenetic Induction of Stress Granules Is Cytotoxic and Reveals the Evolution of ALS‐FTD Pathology,” Elife 8 (2019): 39578, 10.7554/eLife.39578. PMC6426440 30893049

[116] S. Ranganathan , P. Dasmeh , S. Furniss , and E. Shakhnovich , “Phosphorylation Sites are Evolutionary Checkpoints Against Liquid–Solid Transition in Protein Condensates,” Proceedings of the National Academy of Sciences of the United States of America 120, no. 20 (2023): 2215828120, 10.1073/pnas.2215828120. PMC10193986 37155880

[117] R. Wu , D. Zhou , X. Shen , F. Chen , F. Liu , and J. Gu , “Phosphorylation of Trans‐Active Response DNA‐Binding Protein‐of 43 kDa Promotes Its Cytoplasmic Aggregation and Modulates its Function in Tau mRNA Stability and Exon 10 Alternative Splicing,” Journal of Neurochemistry 158, no. 3 (2021): 766–778, 10.1111/jnc.15450. 34107054

[118] D. Sang , T. Shu , C. F. Pantoja , A. Ibáñez de Opakua , M. Zweckstetter , and L. J. Holt , “Condensed‐Phase Signaling Can Expand Kinase Specificity and Respond to Macromolecular Crowding,” Molecular Cell 82, no. 19 (2022): 3693–3711.e10, 10.1016/j.molcel.2022.08.016. 36108633 PMC10101210

[119] W. Peeples and M. K. Rosen , “Mechanistic Dissection of Increased Enzymatic Rate in a Phase‐Separated Compartment,” Nature Chemical Biology 17, no. 6 (2021): 693–702, 10.1038/s41589-021-00801-x. 34035521 PMC8635274

[120] M. Gil‐Garcia , A. I. Benítez‐Mateos , M. Papp , et al., “Local Environment in Biomolecular Condensates Modulates Enzymatic Activity Across Length Scales,” Nature Communications 15, no. 1 (2024): 3322, 10.1038/s41467-024-47435-w. PMC11026464 38637545

[121] M. R. King , K. M. Ruff , A. Z. Lin , et al., “Macromolecular Condensation Organizes Nucleolar Sub‐Phases to Set Up a pH Gradient,” Cell 187, no. 8 (2024): 1889–1906.e24, 10.1016/j.cell.2024.02.029. 38503281 PMC11938373

[122] Y. Dai , Z. Zhou , W. Yu , et al., “Biomolecular Condensates Regulate Cellular Electrochemical Equilibria,” Cell 187, no. 21 (2024): 5951–5966.e18, 10.1016/j.cell.2024.08.018. 39260373 PMC11490381

[123] E. Garner , P. Romero , A. K. Dunker , C. Brown , and Z. Obradovic , “Predicting Binding Regions Within Disordered Proteins,” Genome Informatics Workshop on Genome Informatics 10 (1999): 41–50. 11072341

[124] A. K. Dunker , J. D. Lawson , C. J. Brown , et al., “Intrinsically Disordered Protein,” Journal of Molecular Graphics & Modelling 19, no. 1 (2001): 26–59, 10.1016/s1093-3263(00)00138-8. 11381529

[125] G. Erdős , M. Pajkos , and Z. Dosztányi , “IUPred3: Prediction of Protein Disorder Enhanced With Unambiguous Experimental Annotation and Visualization of Evolutionary Conservation,” Nucleic Acids Research 49, no. W1 (2021): W297–W303, 10.1093/nar/gkab408. 34048569 PMC8262696

[126] J. Jumper , R. Evans , A. Pritzel , et al., “Highly Accurate Protein Structure Prediction With AlphaFold,” Nature 596, no. 7873 (2021): 583–589, 10.1038/s41586-021-03819-2. 34265844 PMC8371605

[127] M. Varadi , S. Anyango , M. Deshpande , et al., “AlphaFold Protein Structure Database: Massively Expanding the Structural Coverage of Protein‐Sequence Space With High‐Accuracy Models,” Nucleic Acids Research 50, no. D1 (2022): D439–D444, 10.1093/nar/gkab1061. 34791371 PMC8728224

[128] P. Brennan , “drawProteins: A Bioconductor/R Package for Reproducible and Programmatic Generation of Protein Schematics,” F1000Research 7 (2018): 1105, 10.12688/f1000research.14541.1. 30210791 PMC6107989

[129] F. Jin and F. Gräter , “How Multisite Phosphorylation Impacts the Conformations of Intrinsically Disordered Proteins,” PLoS Computational Biology 17, no. 5 (2021): 1008939, 10.1371/journal.pcbi.1008939. PMC8148376 33945530

[130] Y. D. Ke , A. van Hummel , C. Au , et al., “Targeting 14‐3‐3θ‐Mediated TDP‐43 Pathology in Amyotrophic Lateral Sclerosis and Frontotemporal Dementia Mice,” Neuron 112, no. 8 (2024): 1249–1264.e8, 10.1016/j.neuron.2024.01.022. 38366598

[131] E. L. Sternburg , L. A. Gruijs da Silva , and D. Dormann , “Post‐Translational Modifications on RNA‐Binding Proteins: Accelerators, Brakes, or Passengers in Neurodegeneration?,” Trends in Biochemical Sciences 47, no. 1 (2022): 6–22, 10.1016/j.tibs.2021.07.004. 34366183

[132] T. R. Suk , C. E. Part , J. L. Zhang , et al., “A Stress‐Dependent TDP‐43 SUMOylation Program Preserves Neuronal Function,” Molecular Neurodegeneration 20, no. 1 (2025): 38, 10.1186/s13024-025-00826-z. 40149017 PMC11951803

[133] E. M. Verde , F. Antoniani , L. Mediani , et al., “SUMO2/3 Conjugation of TDP‐43 Protects Against Aggregation,” Science Advances 11, no. 8 (2025): adq2475, 10.1126/sciadv.adq2475. PMC11844728 39982984

[134] V. Akimov , I. Barrio‐Hernandez , S. V. F. Hansen , et al., “UbiSite Approach for Comprehensive Mapping of Lysine and N‐Terminal Ubiquitination Sites,” Nature Structural & Molecular Biology 25, no. 7 (2018): 631–640, 10.1038/s41594-018-0084-y. 29967540

[135] C. Maurel , A. A. Chami , R.‐A. Thépault , et al., “A Role for SUMOylation in the Formation and Cellular Localization of TDP‐43 Aggregates in Amyotrophic Lateral Sclerosis,” Molecular Neurobiology 57, no. 3 (2020): 1361–1373, 10.1007/s12035-019-01810-7. 31728929

[136] A. Maraschi , V. Gumina , J. Dragotto , et al., “SUMOylation Regulates TDP‐43 Splicing Activity and Nucleocytoplasmic Distribution,” Molecular Neurobiology 58, no. 11 (2021): 5682–5702, 10.1007/s12035-021-02505-8. 34390468 PMC8599232

[137] C. Saunders , P. Rocha‐Rangel , R. Desai , et al., “Citrullination of TDP‐43 Is a Key Post‐Translation Modification Associated With Structural and Functional Changes and Progressive Pathology in TDP‐43 Mouse Models and Human Proteinopathies (p. 2025.02.28.639952),” BioRxiv (2025), 10.1101/2025.02.28.639952.

[138] T. J. Cohen , A. W. Hwang , C. R. Restrepo , C.‐X. Yuan , J. Q. Trojanowski , and V. M. Y. Lee , “An Acetylation Switch Controls TDP‐43 Function and Aggregation Propensity,” Nature Communications 6 (2015): 5845, 10.1038/ncomms6845. PMC4407365 25556531

[139] J. Garcia Morato , F. Hans , F. von Zweydorf , et al., “Sirtuin‐1 Sensitive Lysine‐136 Acetylation Drives Phase Separation and Pathological Aggregation of TDP‐43,” Nature Communications 13, no. 1 (2022): 1223, 10.1038/s41467-022-28822-7. PMC8907366 35264561

[140] S. S. Keating , A. T. Bademosi , R. San Gil , and A. K. Walker , “Aggregation‐Prone TDP‐43 Sequesters and Drives Pathological Transitions of Free Nuclear TDP‐43,” Cellular and Molecular Life Sciences: CMLS 80, no. 4 (2023): 95, 10.1007/s00018-023-04739-2. 36930291 PMC10023653

